# Perceptions of the Muslim religious leaders and their attitudes on herbal medicine in Bangladesh: a cross-sectional study

**DOI:** 10.1186/1756-0500-4-366

**Published:** 2011-09-26

**Authors:** Md Harun-Or-Rashid, Yoshitoku Yoshida, Md Aminur Rashid, Salmun Nahar, Junichi Sakamoto

**Affiliations:** 1 Department of Healthcare Administration, Nagoya University Graduate School of Medicine, Nagoya, Japan; 2 Department of Dermatology, Holy Family Red Crescent Medical College Hospital, Dhaka, Bangladesh; 3 Dhaka Shishu Hospital, Dhaka, Bangladesh

## Abstract

**Background:**

Herbal Medicines (HMs) are playing major roles in the health of the millions of people worldwide. Muslim Religious Leader (MRLs), being an important component of the society with huge influence on it, could contribute a lot to promote HM. This study was aimed at evaluating perceptions of the MRLs, their satisfaction and attitudes towards HM in Bangladesh.

**Methods:**

This cross-sectional study collected data from a purposive sample of 503 MRLs using an interviewer-administered structured questionnaire during December 2010 and January 2011. Main outcome measures included sociodemographic variables, MRLs' preferences in using HMs, their satisfaction and intent to use HMs in the future, and finally MRLs' attitudes toward HM use.

**Results:**

Although two-fifth (40.4%) of the MRLs preferred HM among different form of complementary and alternative medicines, they used orthodox medicine (OM) more frequently than HM in last one year. Majority believed that HM was effective for all age groups (52.6%) and both sexes (74.5%). One-third felt that HM was more effective for chronic diseases, 68.5% felt that it only promotes health, and 40.8% said it keeps them relaxed. About 98.0% of the respondents experienced 'no harm' but 'benefit' from HM; naturally, they were satisfied with HM and were willing to recommend it to others. Urban, older (>40 years), and 'single' respondents were more likely to use HM (gender and education adjusted odds ratios = 1.7 [95% confidence interval, CI = 1.1-2.6], 1.9 [95% CI = 1.3-3.0], and 1.6 [95% CI = 1.2-2.1], respectively). Overall, respondents exhibited very positive attitude towards HM with mean score of 4.2 (range, 3.7-4.6) of a 5-point Likert scale (Score 5 for strongly agree to 1 for strongly disagree).

**Conclusions:**

We report adequate perceptions, satisfaction, and very positive attitudes towards HM among MRLs. Mass media had a significant contribution towards its promotion. If HM are to assume a respected place in the contemporary healthcare, its acceptance among general population needs to be established through incorporating MRLs in the process of HM promotion in Bangladesh.

## Background

Complementary and Alternative Medicine (CAM) includes medical and healthcare systems, practices, and products that are not currently considered as an integral part of orthodox medicine (OM) [1]. In spite of existence of modern and sophisticated healthcare systems in the Western world, millions are turning back to traditional herbal medicines in order to prevent or treat many illnesses, and a reasonable proportion of drugs dispensed in community pharmacies now contain drugs extracted from plants [2]. The higher use of CAM among western society is also reflected in the developing world, indicating that its use is increasing worldwide [3-5]. This may in part reflect a need to fill gaps in conventional Western medical care, including a need for improved therapeutic efficacy, greater cultural or linguistic relevance, and alignment with personal beliefs [6]. It is estimated that 30% to 50% of the general adult population of industrialized nations use one form of CAM or another [7]. The overwhelming majority of people in South Asia including India, Sri Lanka, Bangladesh, and Nepal has been looking up to Ayurveda in its pure form or some of its variations in some form or other, for relief of various ailments [8].

Many studies done in western countries have documented that CAM use is both very common and varies among populations [9]. No doubt that CAM has good values in treating many diseases, which can save lives of many, particularly in the developing countries [10]. In the country like Bangladesh, CAM can play a pivotal role for the country's ever poor public health sector.

In Bangladesh, CAM has long been practiced and it is estimated that 70-75% population of the country still use CAM for management of their health problems of various kinds [11]. Bangladesh is full of medicinal plants which is a potential source of drugs. Drugs from these herbal plants have been widely used even in this modern era. Traditionally it is believed that these herbs have very little side-effects and it cost almost nothing in few cases. So, it can solve the economic problem for the poor [12]. Broadly speaking, four types of CAM are primarily practiced in Bangladesh namely herbal, homeopathy, religious, and magical methods. Based on the existing rich local plant diversity, the tradition of indigenous herbal medicine systems has formed a very important component of the primary healthcare system of Bangladesh [11]. The herbal method of CAM mainly uses plant derived medicines and consists of ayurvedic and unani systems in Bangladesh, of which Ayurveda is the most popular. Both registered and unregistered (locally known as Kabiraj) herbal practitioners are practicing in the country at present. Homeopathy is based on the basic principles of law of similarity, direction of cure, principles of single remedy, the theory of minimum diluted dose, and the theory of chronic disease which is intended to trigger immune system of human body [13]. Religious and magical methods include torture, charms, magic, incantations, religious verses, amulets and rituals like sacrifices, appeasement of evil spirits, etc. Spiritual/Jharfuk is a form of religious method of CAM where religious verses are recited to spiritually heal the ailment of the patient. Although religious methods are widely practiced in Bangladesh, they are not officially recognized as scientific medicines or methods of treatment [14]. Notwithstanding Ayurvedic and Unani medicines are very similar in origin from plants, few people can differentiate these subtle differences. Therefore we intended to know respondents' opinion for herbal medicines (HMs) as a whole, not specifically on Ayurvda or Unani system.

Bangladesh as a Muslim country is highly influenced by religion and Islamic values influence every aspect of people's life. Muslim religious leaders (MRLs) are very influential and trustworthy personnel who are important in shaping social values [15]. Considerable influence of the MRLs can have a positive role in raising the awareness of the society about healthy and nondiscriminating behaviours towards HM. They can stimulate a political response and create a supportive environment in favor of HM. They also can play an important role in spiritual counselling and care for patients and their families.

Despite being in use of CAMs for more than 3000 years, few properly designed trials have scientifically examined the clinical potential and peoples' trust on Herbal and other medications [16]. In Bangladesh also, there has been a great paucity of information on it. Exploring the perceptions and attitudes of MRLs will pave the way to incorporate HM in the healthcare system and to plan culturally appropriate evidence-based strategies to combat diseases and assist health care providers in providing needed treatment and care. Therefore, this study was aimed to explore MRLs' perceptions on HM and their attitudes towards it. This study is a unique one as no study has been carried out till date to determine the perceptions and attitude of the MRLs about HM in the country. This study may be a dependable host of information required for further studies in the relevant fields.

## Methods

### Design and sample

This cross-sectional survey was conducted by face-to-face interview by trained staffs using a structured questionnaire during December 2010 to January 2011. Four data collecting staff were trained on how to collect data from the respondents. Their interviewing skills were assessed through pretesting the questionnaire and interview among 15 MRLs. Participants were chosen purposively from MRLs with educational background in Islamic religion under Madrasah education system. Madrasah education system, a unique system of Islamic religious education, are divided into five distinct levels - Ibtedai (elementary, 1-5 years), Dakhil (secondary, 6-10 years), Alim (higher secondary, 11-12 years), Fajil (undergraduate, 13-14 years), and Kamil (Masters, 15-16/17 years) [17]. These Madrasahs teach all the required modern subjects, such as English, Bengali, Social studies, Maths, Geography etc. in addition to religious subjects [17]. The sample size was consists of 503 MRLs from urban and rural areas of two districts (Dhaka and Brahmanbaria) of Bangladesh. Majority of the participants were involved in teaching in Madrasah and leading prayers in the mosques as 'Imam'. Participants with educational qualification at least Dakhil and age group between 19-60 years were included in this study. Participants who did not use any form of CAM in their life-time were excluded. The subjects were informed that they were free to decline answering any question with which they were not comfortable. Anonymity of their personal identity was preserved. Written informed written consent was obtained from every participant before interview. As this study was not involved in experimental research involving human subjects, ethical approval was exempted.

### Questionnaire

The questionnaire was divided into three parts: Part A contained socio-demographic information of the study participants, such as age, sex, marital status, monthly income, occupation, and self-rating of their health status; Part B contained information about their use of HMs; and part C contained 13 positive questions to assess attitudes of the respondents towards HM. The questionnaire was generated with modification of Patterson and Arthurs' published questionnaire [18]. Then it was validated by three expert reviews for clarity, comprehensibility, and contents. Finally, the questionnaire was translated into Bengali and changes were made to make it understandable for the respondents before data collection in the field and back translated into English. To measure the attitudes of the respondents towards use of HM, we used 13-item scales. The participants were asked to indicate their level of agreement with items on a 5-point Likert scale ranging from 1 = "Strongly disagree" to 5 = "Strongly agree". The total possible score were ranged from 13 (Negative attitude to use HM) to 65 (positive attitude to use HM).

### Statistical analyses

Standard methods of exploratory data analysis (means, standard deviations [SDs], medians, frequencies) were used to depict the distribution of the principal variables. Univariate tests of statistical significance, such as independent sample *t *tests for continuous variables and Chi-square tests for categorical variables were used to draw preliminary conclusions. Inferences from these results were further explored using multivariable logistic regressions to estimate odds ratio (OR) and 95% confidence interval (CI). Level of statistical significance were set at *P *= .05.

MRLs' attitudes toward the use of HM were determined first by computing a total score on the 13-item questionnaire. The mean total score was 54.8 (SD ± 3.7) out of a possible 65. To examine the association of the attitude score with respondents' demographic characteristics, the total score was dichotomized at the median [18]. Accordingly, scores in the range of 0-55 were defined as 'negative attitude' and low likelihood to use HM, whereas scores between 56 and 65 were defined as 'positive attitude' and higher likelihood to use HM.

## Results

Characteristics of the respondents and their preference of HM are summarized in Table 1. Majority of them were urban male with an average age of 37.0 years (SD = 8.6). Most of them had educational background up to 'Dakhil' and 426 (85.5%) were employed as teacher in some Madrasah. Only 3.2% were housewives. They varied in rating of their overall health status as follows: 'Excellent' 15.9% (n = 80); 'Very good' 37.2% (n = 187); 'Good' 23.3% (n = 117); 'So-so' 23.1% (n = 116); and 'Not good' 0.6% (n = 3). Highest number of the respondents informed that they preferred HM (n = 204, 40.4%) followed by 'Spiritual/Jharfuk' which was liked by 31.2%. Homeopathy, although very popular among general population in Bangladesh, only 132 (26.2%) of our respondents liked that.

**Table 1:** Respondents' characteristics and preference of Complementary and Alternative Medicines (CAMs)

Factors	Number (n = 503)	%	Significance
Gender			<.001
Male	450	89.6	
Female	52	10.4	
Age (Years)			
Mean±Standard deviation	37.0 ± 8.6		
Residence			<.001
Urban	389	77.3	
Rural	114	22.7	
Marital status			<.001
Married	404	81.0	
Single	95	19.0	
Education*			<.001
Dakhil	409	82.6	
Alim	59	11.9	
Fajil	13	2.6	
Kamil	14	2.8	
Occupation			<.001
Madrasah teacher	426	85.5	
Business	52	10.4	
Housewife	16	3.2	
Jobless	2	0.4	
Others	2	0.4	
Overall self-health rating			<.001
Excellent	80	15.9	
Very good	187	37.2	
Good	117	23.3	
So-so	116	23.1	
Not good	3	0.6	
Preference of CAMs			<.001
Herbal	204	40.4	
Spiritual/Jharfuk**	157	31.2	
Homeopathy	132	26.2	
Others	10	2.0	

*Madrasah education system in Bangladesh classifies Dakhil, Alim, Fajil, and Kamil as equivalent to Secondary (up to 10 years), Higher Secondary (11-12 years), Undergraduate (13-14 years), and Masters (15-16/17 years of schooling), respectively [17].

**Spiritual/Jharfuk is a form of religious method of complementary and alternative medicine where religious verses are recited to spiritually heal the ailment of the patient.

Responses of the subjects about their perceptions of HM are explained in Table 2. More than half of the respondents believed that HM is effective at any age and only few opined that HM effects at the extreme of ages. Some of them also expressed that HM may be effective for teenage (n = 139, 27.8%) and 20-60 years age group (n = 58, 11.6%). Three-fourth of the respondents believed that HM is equally effective for both sexes and there was no substantial differences of opinion about disease specificity of HM; however, 341 (68.5%) felt that HM promotes health rather curing or preventing diseases directly.

**Table 2:** Perceptions about Herbal Medicine (HM) use among respondents (n = 503)

Characteristics	Number	%	Significance
HM is effective for age group -			<.001
Up to 12	22	4.4	
13-19	139	27.8	
20-60	58	11.6	
>60	18	3.6	
All ages	263	52.6	
HM is effective for -			<.001
Only male	99	19.8	
Only female	28	5.6	
Both male and female	372	74.5	
Diseases best treated by HM -			<.001
Common diseases	119	23.9	
Complicated diseases	83	16.7	
Acute diseases	133	26.7	
Chronic diseases	163	32.7	
Mode of effect of HM -			<.001
Prevention of disease	56	11.2	
Treatment of diseases	97	19.5	
Promotion of health	341	68.5	
Others	4	0.8	
How HM works?			<.001
Eradicate disease	81	16.3	
Improve body defence	192	38.6	
Keeps relax	203	40.8	
Remove bad effect of OM*	17	3.4	
Cures symptoms only	4	0.8	

*OM = Orthodox medicine

Table 3 illustrates that a quarter of the respondents used OM as their first choice followed by HM, half of them used both together to get synergistic effects. Nearly all of the respondents were satisfied using HM as they got benefit without any harmful side-effects; accordingly, they were motivated to recommend HM to others. More than half of the respondents thought that they would use OM in case the treatment cost would be same for both choices. Four hundred forty-nine (90.5%) respondents recommended for more initiatives from the government side to promote HM in Bangladesh.

**Table 3:** Satisfaction on Herbal Medicine (HM) use among respondents (n = 503)

Characteristics	Number	%	Significance
Priority of use			<.001
First HM then OM*	145	28.9	
First OM then HM	127	25.3	
Both together	229	45.7	
Did you get benefit from HM?			<.001
Yes	486	97.4	
No	13	2.6	
Did you get harm from HM?			<.001
Yes	7	1.7	
No	492	98.6	
Are you satisfied with HM?			<.001
Very satisfied	244	49.1	
Satisfied	249	50.1	
Upset	4	0.8	
Will you recommend HM to others?			<.001
Yes	477	97.9	
No	10	2.1	
From where do you collect HM?			.025
Friends	121	24.3	
Relatives	120	24.1	
Herbal health workers	105	21.1	
Shop	152	30.5	
If treatment cost is same, which one will you choose?			<.001
OM	290	58.6	
HM	205	41.4	
Does anybody help you using HM?			<.001
Yes	369	74.1	
No	129	25.9	
Government should take more initiatives to promote HM			<.001
Yes	449	90.5	
No	4	0.8	
Existing initiatives are enough	43	8.7	

*OM = Orthodox medicine

Distance of HM clinic/hospitals was a bit far than OM clinic/hospitals (*P *<.001). Annual expenditure on HM was also significantly lower than OM, although they mentioned that per-visit expenditure was cheaper for HM than OM (*P *<.001). More than half of the respondents used HM 1-2 times a year and 49.1% (244) of the respondents used OM 3-4 times a year. Overall use of OM was higher than HM (Table 4).

**Table 4:** Comparative use of Herbal and Orthodox Medicine in last one year

Frequency of use	Herbal medicine		Orthodox medicine		Significance
			
	Number	%	Number	%	
Never	3	0.6	0	0.0	<.001
1-2	295	58.9	59	11.9	
3-4	152	30.2	244	49.1	
5-6	48	9.6	185	37.2	
>6	3	0.6	9	1.8	

**Figure 1:**
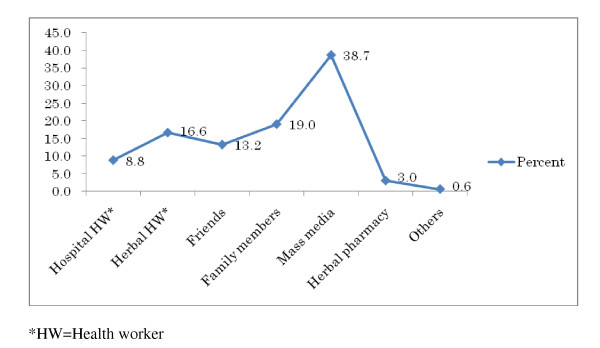
Different sources of information about Herbal medicine (n = 503)

Figure 1 describes different sources of information of HM obtained by the respondents. Leading source of information was mass media (n = 193, 38.7%) followed by family members (n = 95, 19.0%), Herbal health workers (n = 83, 16.6%), friends (n = 66, 13.2%), and hospital health workers (n = 44, 8.8%).

Details of the attitudes of the respondents towards HM are depicted in Table 5. MRLs had a very positive attitude with an average score of 4.2 (minimum, 3.7 and maximum, 4.6) from a 5-point Likert scale. The lowest score for attitude were 3.7 on teacher's role to influence students towards HM. Motivation to use HM by mass media, like Radio, Television etc. also showed a greater contribution in their HM uses. 'Urban' 'older (>40 years)', and 'single' respondents were almost twice more likely to use HM. After adjusting for gender and education their likelihood of using HM remained high (adjusted ORs = 1.7 [95% CI = 1.1-2.6], 1.9 [95% CI = 1.3-3.0], and 1.6 [95% CI = 1.2-2.1], respectively).

**Table 5:** Attitudes of the respondents on Herbal Medicine (HM)

Items	SA	A	NC	D	SD	Average
						
	N(%)	N(%)	N (%)	N(%)	N(%)	score
HM providers give good information on maintaining a healthy lifestyle (n = 502)	207 (41.2)	281 (56.0)	11 (2.2)	0 (0.0)	3 (0.6)	SA 4.4
There are less side effects when taking natural remedies like HM (n = 501)	290 (57.7)	175 (34.9)	29 (5.8)	6 (1.2)	1 (0.2)	SA 4.5
HM involves natural plant formulas which are more healthy than taking drugs given by the medical doctor (n = 501)	137 (27.3)	257 (51.3)	66 (13.2)	25 (5.0)	16 (3.2)	A 4.0
People would be more likely to use HM if there were more HM clinics (n = 500)	203 (40.6)	234 (46.8)	43 (8.6)	4 (0.8)	16 (3.2)	SA 4.2
HM builds up the body's own defences and promotes self-healing (n = 501)	249 (49.7)	235 (46.9)	14 (2.8)	2 (0.4)	1 (0.2)	SA 4.5
The more knowledge a person has about HM, the more likely he/she is to use it (n = 503)	247 (49.1)	234 (46.5)	17 (3.4)	2 (0.4)	3 (0.6)	SA 4.4
Parent(s) and family can influence a young adult's HM use by exposing them to it (n = 500)	192 (38.2)	249 (49.6)	32 (6.4)	16 (3.2)	13 (2.6)	SA 4.1
People can be influenced to use HM if their friends are using it (n = 503)	172 (34.2)	239 (47.5)	52 (10.3)	14 (2.8)	26 (5.2)	A 4.0
Young adults are more likely to use HM if coaches and teachers discuss it with them	129 (25.7)	208 (41.4)	107 (21.3)	21 (4.2)	37 (7.4)	A 3.7
People who believe in the physical, mental, and spiritual aspects of health are more likely to use HM (n = 498)	130 (26.1)	232 (46.6)	79 (15.9)	21 (4.2)	36 (7.2)	A 3.8
Those who fear the discomfort of treatments from medical doctors are more likely to use HM (n = 500)	140 (28.0)	293 (58.6)	38 (7.6)	16 (3.2)	13 (2.6)	SA 4.1
Taking HM therapies are not harmful (n = 503)	325 (64.6)	170 (33.8)	6 (1.2)	2 (0.4)	0 (0.0)	SA 4.6
People are mostly motivated to use HM by Radio, Television, and mass media campaign (n = 499)	236 (47.3)	261 (52.3)	0 (0.0)	2 (0.4)	0 (0.0)	SA 4.5
Overall	205 (40.7)	237 (47.1)	38 (7.6)	10 (2.0)	13 (2.5)	4.2

SA = Strongly agree, A = Agree, NC = No comments, D = Disagree, SD = Strongly disagree

## Discussion

To the best of our knowledge, this is the first study focusing on the perceptions of HM use and attitudes towards it among the MRLs in Bangladesh. The key findings of this study were: 1) majority of the MRLs preferred HM over other CAMs; 2) higher satisfaction of MRLs towards HM and their willingness to recommend it to others in their family, friends and so on; and 3) their positive attitudes to HM.

Although there had been a notable advancement in the field of HM in the Western countries, Bangladesh is still struggling to give HM a respectable position in the mainstream healthcare system. Following independence of Bangladesh, the Board of Unani and Ayurvedic Systems of Medicine, Bangladesh was restructured with Bangladesh Unani and Ayurvedic Practitioners Ordinance of 1972 [13]. Since then, several steps had been taken to strengthen HM in Bangladesh. However, to date there appears to be no harmonized system of overseeing the practice of HM in Bangladesh. But efforts to formulate a law to recognize, coordinate and regulate the practice of HMs are underway.

We found that preference for HM use was very high among the respondents which are consistent with the findings of other published reports of similar design [7]. However, their health seeking behaviour contradicts their saying that 'they prefer OM - if the treatment cost were same'. This discrepancy may be explained by understanding that the treatment sought is determined by the ailment one is suffering from. For easily diagnosed ailments and for those with well-established cures, e.g. malaria, they prefer to go to OM practitioners (OMPs), but for difficult to diagnose and chronic illnesses they seek the services of HM practitioners (HMPs) [19]. Affordable cost of HM may be another reason of such discrepancy. In Bangladesh, HM use is still limited to the middle- and low-income groups of general population. Because of lack of a health insurance system, patients must pay treatment costs out of their pocket. Middle- and low-income people naturally choose to use HM first, not because of their choice, but because HM is cheap, more closely corresponds to their ideology, and is less paternalistic than OM [13]. However, preference of HM may be higher in our study than in general population, especially respondents with higher income chose HM more than those with lower income (*P *= .04). Religious affiliations may also appear to influence the pattern of use of HM in every population [1]. This is also reflected in our result that 'Spiritual/Jharfuk' was the second popular CAM among them. Religious influences over HM is also reported by Ezeome *et al. *in their study saying that the high proportion of Catholics among their study population explains the increased prevalence in the use of religious relics and items such as black stone, olive oil, and mustard seed [1]. Other explanations of liking HM but using OM may be: their less confidence on the scientific basis of HM; OM is more effective than HM; OMPs make better diagnoses of illnesses; the people are more familiar with OM; and some charlatans charge a lot of money to cure simple ailments [19]. There are not enough scientific evidences which can surely indicate that scientific basis of HM is beyond doubt and comparable with modern medicines like Allopathy [2]. Over- and inappropriate use of HM is also rampant. There is a obvious shortage of skilled human resources in the field of HM as it was reflected from the report of Ashraf et al. [20]. Still now after a long time of Ashraf et al.'s study, there has been no remarkable improvement in the HM system. Quackery in the field of HM is also quite common. Even a layman without proper know-how can become an HMP to look for additional earnings. This type of practice may pose serious threats to public health often by erroneous diagnosis and incorrect treatment using wrong or fake medications [11].

Surprisingly, we did not find any differences in preferences of HM by gender and level of education which has been observed in many other studies [7,21,22]. This could be subject of sampling or reflect an aspect of preferences by MRLs. However, urban and older participants showed significantly higher preference to HM. Similar findings were reported by many other studies [21,22]. It is expected that increased age is commonly associated with greater health ailments, especially chronic diseases [7,23-25], and higher familiarity with traditional methods, and reluctance over OM [6].

It is well known that South Asian countries, like Bangladesh, India, Nepal, and Sri Lanka bear many cultural and religious practices in common. Especially they are highly influenced by religious rituals in almost every spheres of their life including medication. A major portion of the CAMs in these countries are dominated by use of religious practices. For example, the herb Tulsi also called holy basil (sometimes spelled "Tulasi") has been widely known for its health promoting and medicinal value for thousands of years. Commonly called sacred or holy basil, it is a principal herb of Ayurveda, the ancient traditional holistic health system of India. Holy basil is known as "The Incomparable One", "The Mother Medicine of Nature", and "The Queen of Herbs" [26]. Dhami-jhankri, Pandit-lama-gubhaju-pujari and Jyotishi are the leading 'Faith healers' in Nepal having huge influence on CAM practice in Nepal [27]. Thereby, it is expected that they are also motivated to a great extent by the religious leaders before deciding to use HM in their sickness. Importantly, religious influences are abundant in all classes of population in Bangladesh. For any kind of sickness or other issues people always rely on the suggestion of the MRLs. Again because of faith-related matters, these MRLs prefer to use something which has more natural value like herbs in HM. Their feelings were well reflected in our study where we found that they were very satisfied with HM and they want to recommend others to use HM. This may not be because their higher satisfaction with all aspects of diseases and their treatment with HM, rather because of their belief that HM is less harmful than OM. Given them enough evidence that HM is equally reliable in terms of the scientific basis with OM could convince them more towards HM. Whatever may be the reason, it is a very meaningful observation to promote HM farther in Bangladesh. In addition, they stated very positive attitudes towards questions related to HM which can add momentum in their efforts to promote HM.

We found mass media play the leading role in promoting HM in Bangladesh followed by family members and friends which was in line with other published report [11]. It is evidenced that aggressive advertisements of HM on various media including newspapers have resulted in increased sales all over the world [2]. However, exaggerated and superfluous advertisement in the mass media has also lost its faith especially from the educated people. Very often, less educated and low-income people are deceived out of this advertisement which is again put negative impact on the HM. The advertisements in the newspapers promise to prevent and even cure incurable conditions - AIDS (acquired immunodeficiency syndrome), cancer, heart disease, diabetes, arthritis, multiple sclerosis and so on [11]. Very often they claim that HM can give absolute guarantee of cure even when conventional medicine fails. Consequently, this weapon of attracting people towards HM is getting blunt. At this crucial point, it is necessary to find some alternative to return faith of people towards HM.

Our findings of higher satisfaction and positive attitude towards HM among MRLs could show us an important direction towards promotion of HM in Bangladesh. Nurturing adequate perception about HM, as expressed by the MRLs, lead to attainment of positive attitude towards it. Having such positive attitudes can persuade people to use and advocate HM to the community people. In addition, these MRLs need more exposure with training and orientation about HM to be enough knowledgeable on the scientific basis of HM and its safety. Only then, it may be convenient and cost-effective to seek help from the MRLs in the progression of HM development in Bangladesh. As such, the mass media could regain their lost faith from the people if they can incorporate MRLs to promote HM.

Several limitations of our study need to be pointed. As Ayurveda is an important component of herbal medicines and had its roots from Hindu scripture Samaskrutham, a potential conflict of religious belief among our respondents cannot be ruled out. Despite this possibility, Ayurveda was the most popular among all forms of CAMs in Bangladesh which could justify the rationale of considering these MRLs in HM promotion in Bangladesh. Our questionnaire was developed after review and modification of existing studies, which were from industrialized Western countries. Although we made substantial changes to make it relevant to our local environment, possibility of not conveying important insights on the Bangladeshi respondents exists. A properly designed qualitative study can provide such insights. We included only a small sample of MRLs which might be a threat to the generalizability of the result. Finally, given the long history of HM in Bangladesh, and its intimate integration into daily life, some participants may have been employing Herbal concepts and methods, such as use of specific medicinal herbs or spices in cooking, without recognizing it as such, and therefore underestimation of HM use cannot be ruled out. Despite these shortcomings, we believe this study gives a reliable picture of HM use among MRLs in Bangladesh.

## Conclusions

This study reports adequate perceptions about use, effectiveness, safety, availability, and affordability of HM among MRLs. They were highly satisfied with HM and ready to recommend others. Mass media like Radio, Television also had enormous impact on its promotion. Taken together, these findings suggest the need to educate MRLs about the scientific basis of HM and to utilize them generously to solicit information about use of HM. Government's recognition and its implementation in the healthcare machineries are important to promote HM in Bangladesh. Nevertheless, if HM are to assume a respected place in the contemporary healthcare, its acceptance among general population needs to be established through incorporating MRLs in the progression of HM promotion in Bangladesh. Future empirical research with larger sample including leaders of all sects could precisely formulate next step to establish HM in Bangladesh.

## Competing interests

The authors declare that they have no competing interests.

## Authors' contributions

All authors contributed to study concept and design. MHOR conducted the literature review, performed the analyses and prepared major portions of the draft manuscript, and edited the final version of the manuscript. MAR and SN contributed in obtaining the data and helping with the analysis. YY and JS contributed to the design of the study and critical review of the final manuscript. All authors interpreted the data, revised the article critically for important intellectual content and approved the final version.
